# Evaluation of pulse oximeter derived photoplethysmographic signals for obstructive sleep apnea diagnosis

**DOI:** 10.1097/MD.0000000000006755

**Published:** 2017-05-05

**Authors:** Yan Li, He Gao, Yan Ma

**Affiliations:** a Aerospace Sleep Medicine Center, Airforce General Hospital of PLA, Beijing, China; b Division of Interdisciplinary Medicine and Biotechnology, Department of Medicine, Beth Israel Deaconess Medical Center, Harvard Medical School, Boston, MA, USA.

**Keywords:** agreement, obstructive sleep apnea, photoplethysmography, polysomnography, portable monitors, pulse oximeter, sleep

## Abstract

High prevalence of obstructive sleep apnea (OSA) has increased the demands for more convenient and accessible diagnostic devices other than standard in-lab polysomnography (PSG). Despite the increasing utility of photoplethysmograph (PPG), it remains understudied in underserved populations. This study aimed to evaluate the reliability of a standard pulse oximeter system with an automated analysis based on the PPG signal for the diagnosis of OSA, as compared with PSG derived measures.

Consecutive out-patients with suspect OSA completed a PPG monitoring simultaneous with an overnight in-lab standard PSG. Forty-nine OSA patients (38 males, age 43.5 ± 16.9 years, BMI 26.9 ± 0.5 kg/m^2^) were included in this study. Automated analyses were based on PPG and oximetry signals only. The PPG calculated measures were compared with PSG derived measures for agreement tests.

Respiratory events index derived from PPG significantly correlated with PSG-derived apnea–hypopnea index (*r* = 0.935, *P* < .001). The calculation of total sleep time and oxygen desaturation index from PPG and PSG also significantly correlated (*r* = 0.418, *P* = .003; *r* = 0.933, *P* < .001, respectively). Bland–Altman plots showed good agreement between the PPG and the PSG measures. The overall sensitivity and specificity of PPG are good, especially in moderate and severe OSA groups.

The tested PPG approach yielded acceptable results compared to the gold standard PSG among moderate to severe OSA patients. A pulse oximeter system with PPG recording can be used for the diagnosis or screening of OSA in high risk population.

## 1 Introduction

As a major form of sleep-disordered breathing (SDB), obstructive sleep apnea (OSA) is estimated to have a prevalence ranging from 9% to 38% among the general population.^[1]^ OSA causes or contributes to sleep fragmentation, hypoxemia, hypercapnia, nocturia, morning headaches, excessive daytime sleepiness, and mood change.^[2]^ It also increases the risks of cardiovascular disease,^[3,4]^ neurocognitive dysfunction,^[5,6]^ diabetes,^[7,8]^ gastroesophageal reflux disease,^[9,10]^ or even sudden death.^[11]^ Therefore, early diagnosis and treatment of OSA can dramatically reduce the risk of morbidity and mortality in associated diseases.^[12–16]^

Although attended polysomnography (PSG) in sleep labs has been considered as a gold standard diagnostic measurement for SDB,^[17,18]^ it requires the placement of multiple sensors during an overnight stay in the laboratory, involves hours of manual scoring, and is difficult to rapidly expand services. The development of a readily obtained, surrogate marker of OSA has important clinical implications, and the demands on such screening devices for simple and cost-effective OSA diagnosis has been increasing.^[19]^ Although the overall prevalence of OSA is not necessarily higher in Asians, OSA may be more prevalent in patients with equivalent body mass indices because of craniofacial differences.^[20,21]^ It has been reported that OSA and its comorbid are underdiagnosed in China.^[22]^ Therefore, such demands are especially highlighted in China, where the large number of population significant outweighs its limited medical resources.^[23–27]^

Photoplethysmography (PPG) is one of the existing techniques that realize portable monitoring or home sleep studies. PPG is a simple and low-cost optical technique that can be used to detect blood volume changes in the microvascular bed of tissue,^[28]^ and is often obtained by using a pulse oximeter, noninvasively to make measurements at the skin surface. In recent years, PPG technology has been used in a wide range of commercially available medical devices, especially those for measuring oxygen saturation and detecting apneic events.^[28]^ However, the relative paucity of clinical studies of PPG applications was conducted in Caucasian populations.^[29–31]^ In this study, we aim to evaluate the sensitivity and specificity of a portable PPG-based sleep monitor (Morpheus Ox), and assess the correlations of the PPG-based respiratory events index (PPG-REI) and conventional PSG-based apnea–hypopnea index (PSG-AHI) in Chinese.

## 2 Methods

### 2.1 Subject recruitment

All recruited subjects were out-patients referred to the Aerospace Sleep Medicine Center at Airforce General Hospital of PLA, for the evaluation of suspected OSA. Inclusion criteria were: age 18 to 80 years and voluntarily wear a PPG-based monitor in addition to standard overnight diagnostic PSG study. Exclusion criteria included congestive heart failure, hypoventilation syndromes (eg, chronic obstructive pulmonary disease), central sleep apnea syndrome, neuromuscular disease, previously diagnosed OSA, and currently under interventions such as positive airway pressure or oxygen therapy. Data were excluded if any of the PSG or oximetery recording was less than 2 hours. Fifty-one subjects were recruited and completed an overnight PPG monitoring simultaneously with gold standard full PSG, while 2 of them were excluded because of the data length is less than 2 hours. Forty-nine subjects were included in the study (38 male, age 43.5 ± 16.9 years, BMI 26.9 ± 3.7 kg/m^2^).

The study was approved by the Institutional Review Board of the Airforce General Hospital, and we confirm that all experiments were performed in accordance with relevant guidelines and regulations. All subjects were acknowledged of the study protocol, and provided written informed consent prior to participating in the study. All subjects have provided written informed consent prior to participating in the study.

### 2.2 Study design and data acquisition

#### 2.2.1 Polysomnography protocol

All subjects underwent standard overnight PSG in the sleep laboratory. Sleep studies were performed using the Compumedics E-Series (PSG Online 3, Compumedics Ltd, Abbotsford, Australia). PSG montages are placed according to current American Academy of Sleep Medicine (AASM) recommendations,^[32]^ including 6 electroencephalography channels (F4-M1, C4-M1, O2-M1, F3-M2, C3-M2, and O1-M2), 2 electromyogram (EOG) channels (ROC-M2, LOC-M2), submental electromyography (EMG), bilateral anterior tibialis EMG, electrocardiogram (ECG), dual thoracoabdominal respiratory inductance plethysmography (RIP) belts, finger pulse oximetry, a vibration snore sensor, nasal pressure airflow, oronasal thermocouple, and body position.

#### 2.2.2 PSG scoring

PSG studies were manually scored by registered polysomnographic technologists (RPSGT) according to AASM recommendations (AASM Manual for the Scoring of Sleep and Associated Events, version 2.3).^[32]^ An apneic event was defined when all of the following criteria are met: there is a drop in the peak signal excursion by ≥90% of pre-event baseline using an oronasal thermal sensor; the duration of the ≥90% drop in sensor signal lasts at least the minimum duration as specified by obstructive, mixed, or central apnea duration criteria; and the event meets respiratory effort criteria for obstructive, central, or mixed apnea. Hypopnea is scored if all of the following criteria are met: the peak signal excursions drop by ≥30% of pre-event baseline using nasal pressure or an alternative hypopnea sensor; the duration of the ≥30% drop in signal excursion is ≥10 seconds; and there is a ≥3% oxygen desaturation from pre-event baseline or the event is associated with an arousal.

PSG derived AHI (PSG-AHI) was defined as the total number of apnea and hypopnea events per hour of sleep, and OSA diagnosis is based on the International Classification of Sleep Disorders, Third Edition (ICSD-3).^[33]^ Mild, moderate, and severe OSA were defined using PSG-AHI cut-off points of 5, 15, and 30, respectively.^[34]^ Oxygen desaturation index (ODI) was calculated based on the defined events when there is a ≥3% oxygen desaturation from pre-event baseline.^[32]^

#### 2.2.3 Pulse oximeter photoplethysmograph

**Figure 1:**
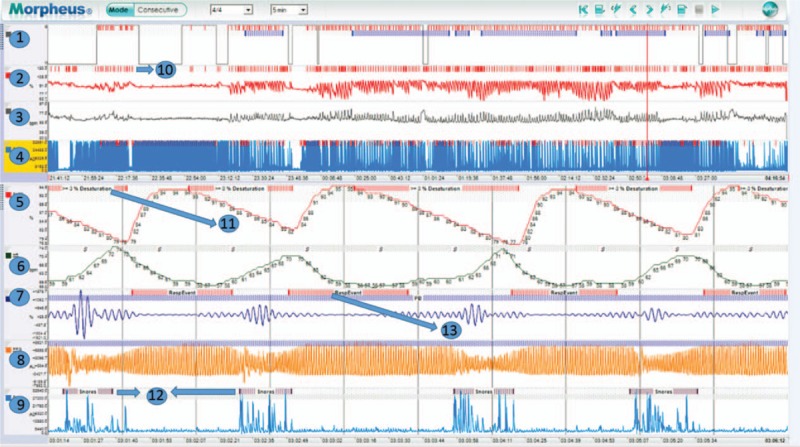
Screen shot for physiological signals and automatic detections of events presented in the Morpheus Ox automatic analysis system. The upper panel shows the overnight trends and sleep structure. 1: sleep stages; 2: SpO_2_; 3: heart rate; 4: snore; 10: automatic detections for oxygen desaturation events. The lower panel shows biological signals in a 5-minute window. 5: SpO_2_; 6: heart rate; 7: PDR, photoplethysmography-derived respiration; 8: PPG, photoplethysmography; 9: snore; 11: 3% desaturation; 12: automatic detections for snoring; 13: automatic detections for respiratory events. PDR = photoplethysmography-derived respiration, SpO_2_ = oxygen saturation as measured by pulse oximetry.

A portable pulse oximeter, PPG-based sleep monitoring device, the Morpheus Ox (WideMed Ltd., Herziliya, Israel) is used to simultaneously record oxygen saturation signals at the finger tip. Recorded physiological data were stored in the device and later downloaded to the computer system, where the software can retrieve the data and generate the waveforms. The software is able to automatically detect respiratory events and calculate respiratory events (REI), total sleep time (TST), oxygen saturation, ODI, and identify sleep/wake epochs from the PPG signal (Fig. 1). By analyzing the PPG for baseline variations, envelope, and rate, PPG-derived respiration (PDR) waveforms can be extracted.^[30]^ The signal processing application can detect the apnea episodes using the PDR amplitude changes that are correlated with oxygen desaturation at level of 3% or 4%. In order to compare with PSG-based sleep outcomes, the 3% oxygen desaturation rule is set for automatic analysis.

### 2.3 Statistical analyses

Data were double entered into a database and checked for outliers and normality of the distribution. Statistical analyses were performed using SPSS version 22 (IBM SPSS Statistics, NY) and MedCalc version 16.2.0 (MedCalc Software BVBA, Oostende, Belgium). For power calculation, we assumed α = 0.05, β = 0.10, 2-sided significance testing, and 10% study dropout. Under these assumptions, the study would need to enroll at least 40 patients. Demographic and sleep data were described as mean ± standard deviation (SD) for normal distributed data, or median (interquartile range [IQR]) for skewed data. Sleep parameters derived from both devices were compared using paired *t* tests or Wilcoxon signed rank tests for normally and nonnormally distributed continuous data, respectively. Consistency analysis included calculation of sensitivity, specificity, positive and negative likelihood ratios (LR+, LR−), negative predictive value (NPV), positive predictive value (PPV), agreement, and Kappa test by SPSS using the PSG as the referenced standard. Sensitivity is defined as true positive divided by positive samples and specificity is defined as true negative divided by negative samples. Positive LRs are defined as sensitivity/(1 − specificity) and negative LRs are defined as (1 − sensitivity)/specificity. NPV value is calculated by true negative divided by the sum of true negative and false negative and PPV true positive divided by the sum of true positive and false positive. Bland–Altman plots are also used for agreement analysis by MedCalc. An ROC curve was generated to show the relationship between PPG-REI and PSG-AHI. All statistical tests were 2-tailed and were considered significant if *P* < .05.

## 3 Results

### 3.1 Subject characteristics

Demographic characters of the included subjects were shown in Table 1. As indicated by PSG-AHI, there were 14 (28.6%) mild OSA, 13 (26.5%) moderate OSA, and 16 (32.7%) severe OSA. Higher BMI was found in severe OSA subjects, although the difference was not significant (*P* = .059). Total sleep time derived from PPG-based monitor (324.0 ± 98.4 minutes) is reported to be shorter compared with that from PSG (380.6 ± 74.0 minutes), and the difference was significant (*P* < .001).

**Table 1:**
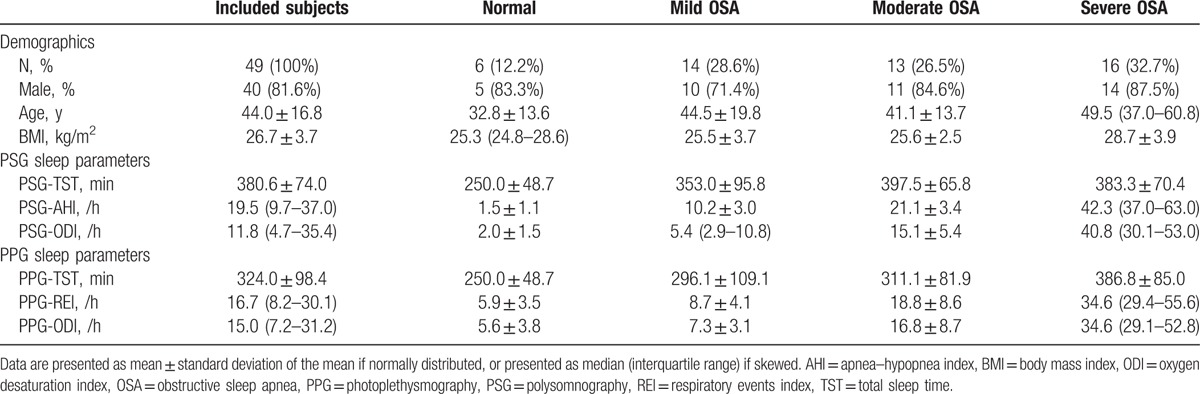
Characteristics of the study cohort.

### 3.2 Correlation between PSG- and PPG-based measures

**Figure 2:**
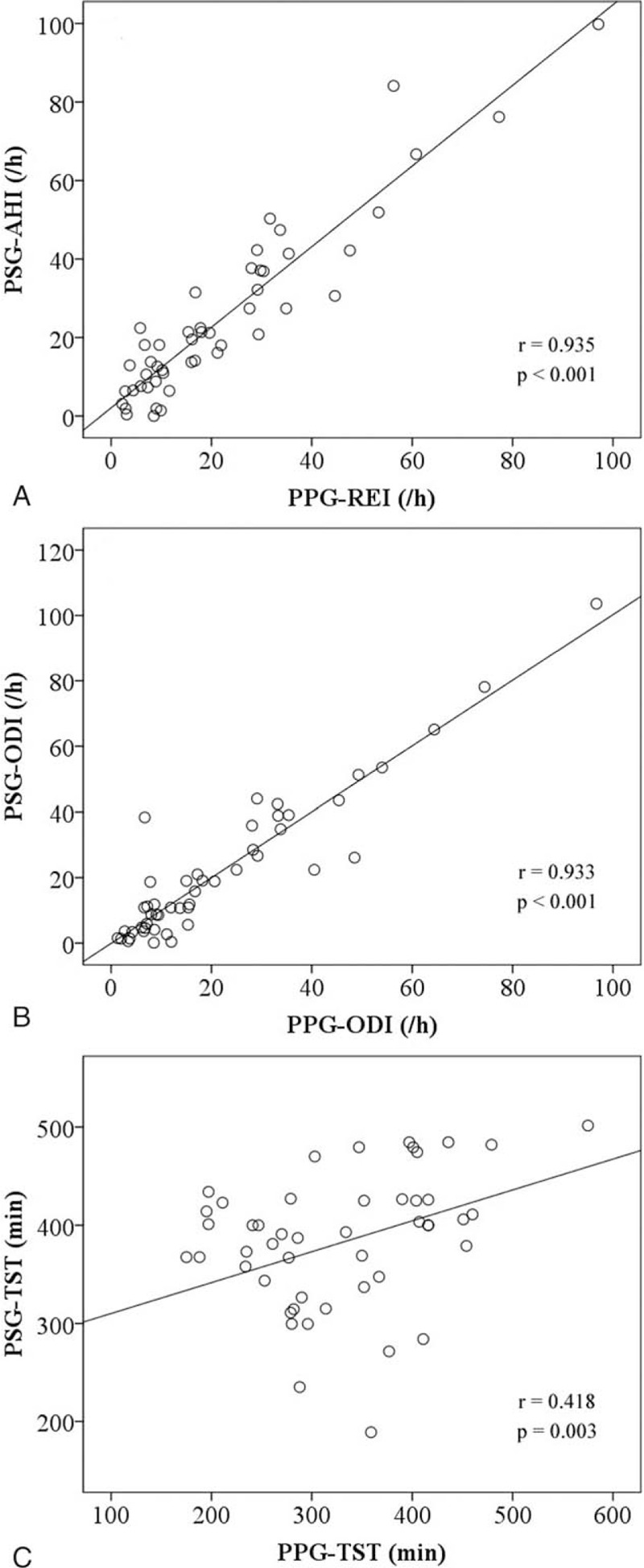
Correlation of PPG- and PSG-based measures. (A) The correlation coefficient (*r*) of PPG-REI and PSG-AHI is 0.935 (*P* < .001); (B) the correlation coefficient (*r*) of PPG-ODI and PSG-ODI is 0.933 (*P* < .001); and (C) the correlation coefficient (*r*) of PPG-TST and PSG-TST is 0.418 (*P* = .003). AHI = apnea–hypopnea index, ODI = oxygen desaturation index, PPG = photoplethysmography, PSG = polysomnography, REI = respiratory events index TST = total sleep time.

**Figure 3:**
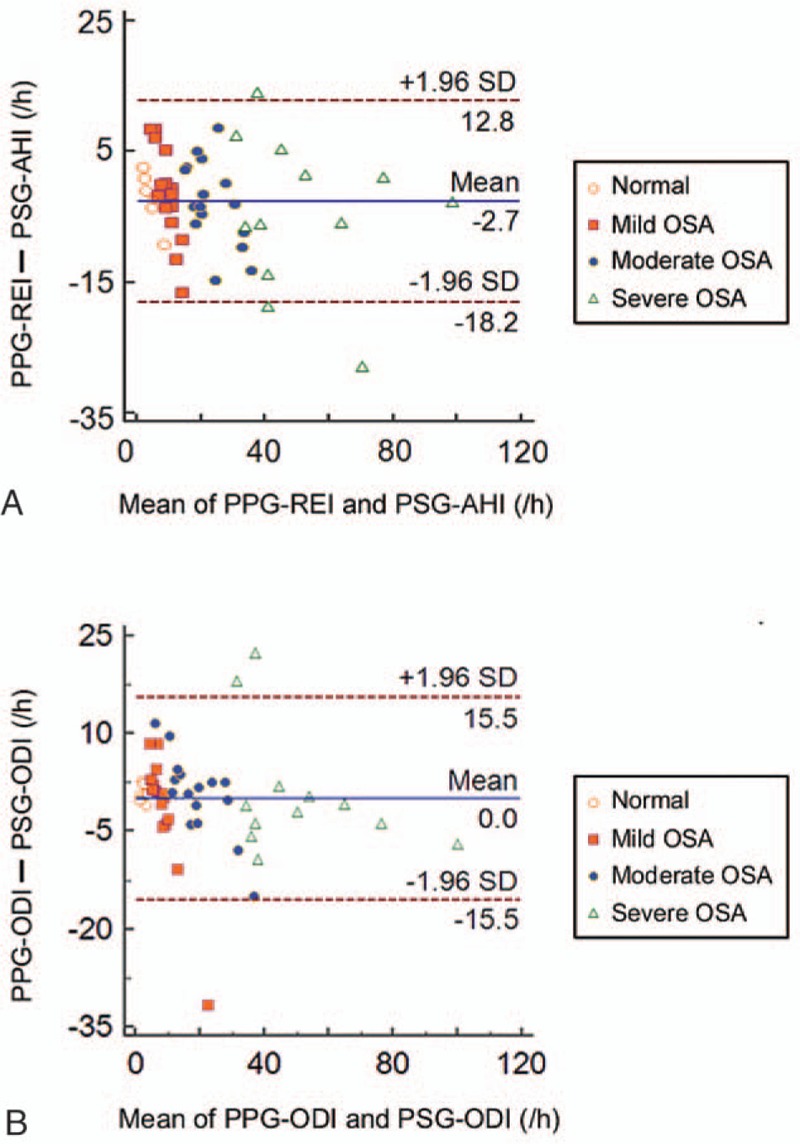
Bland–Altman plot for agreement of PPG and PSG. (A) Agreement of PPG-REI and PSG-AHI; and (B) agreement of PPG-ODI and PSG-ODI. AHI = apnea–hypopnea index, ODI = oxygen desaturation index, PPG = photoplethysmography, PSG = polysomnography, REI = respiratory events index.

The correlations of PPG measures and gold standard PSG measures were shown in Fig. 2. Significant correlations were found between PPG-REI and PSG-AHI (*r* = 0.935, *P* < .001), PPG-ODI and PSG-ODI (*r* = 0.933, *P* < .001), as well as PPG-TST and PSG-TST (*r* = 0.418, *P* = .003). After adjusting for age, gender, and BMI, the parameters were still significantly correlated (PPG-REI and PSG-AHI, *r* = 0.860, *P* < .001; PPG-ODI and PSG-ODI, *r* = 0.926, *P* < .001; and PPG-TST and PSG-TST, *r* = 0.399, *P* = .007). The Bland–Altman plots (Fig. 3) showed good agreement between the PPG and the PSG measures, with most estimates falling within 2 standard deviations of the mean.

### 3.3 Diagnostic accuracy of the PPG measures

**Figure 4:**
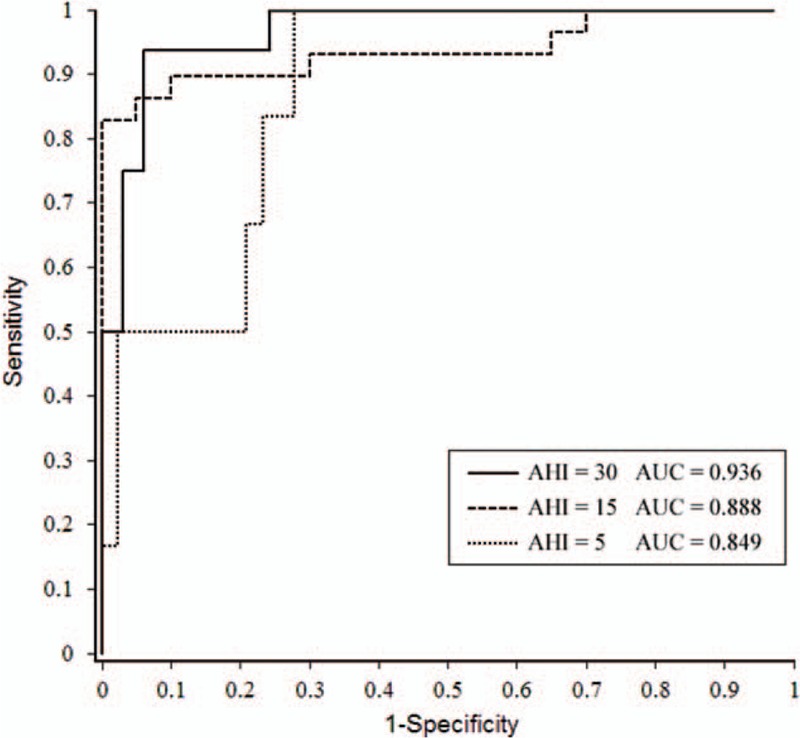
The receiver operating characteristic curves for PPG-REI versus PSG-AHI. AHI = apnea–hypopnea index, PPG = photoplethysmography, PSG = polysomnography, REI = respiratory events index.

The performance of PPG algorithm regarding the diagnostic accuracy of PPG-REI compared with PSG-AHI was evaluated by sensitivity, specificity, PPV and NPV, agreement, positive and negative LRs, and Kappa value (Table 2). For mild sleep apnea, PPG indicated a sensitivity of 95.3%, but a specificity of 50.0%. For moderate and severe sleep apnea, PPG presented a sensitivity of 89.7% and 68.8%, while a specificity of 90.0% and 97.0%, respectively. Overall, the sensitivity is the highest in mild sleep apnea and specificity is highest in severe cases. By ROC analysis (Fig. 4), the 3 curves were shown with the AHI cut-off points set at 5, 15, and 30 events/h, and area under the curve is 0.849, 0.888, and 0.936, respectively. The cut-off points to separate normal, mild, moderate, and severe sleep apnea were found to be 9.9, 16.7, and 27.6 events/h, respectively.

**Table 2:**
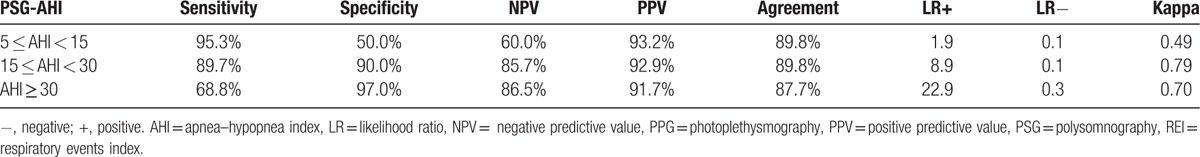
Diagnostic accuracy of PPG-REI compared to PSG-AHI.

## 4 Discussion

This study included 49 subjects for an overnight PPG monitoring simultaneously with gold standard full PSG sleep study. Significant correlations were found between the major measures from the 2 approaches, including PPG-REI and PSG-AHI, PPG-ODI and PSG-ODI, as well as PPG-TST and PSG-TST. The performance of PPG algorithm regarding its diagnostic accuracy of PPG-REI was compared with PSG-AHI. The overall sensitivity, specificity, positive, and NPVs indicated a good accuracy. The PPG-based diagnostic accuracy in moderate and severe OSA patients showed an agreement of 89.8% and an LR+ of 8.9, suggesting that the performance of PPG is consistent with the currently recommended criteria for an acceptable portable monitoring device to confidently “rule-in” OSA (AHI ≥15 events/h) in a high pretest probability clinic population.^[17]^ These findings indicate that a PPG-based monitor (standard pulse oximeter system) for the screening diagnosis of OSA has a high pretest probability.

AHI and blood oxygen saturation derived from PSG-based sleep study are the currently used standard for OSA diagnosis.^[35]^ So, the diagnostic ability of a sleep monitoring device is often evaluated by the accuracy of the AHI or REI, along with and oxygen desaturation. PPG provides valuable information about the cardiovascular system, including cardiac synchronous changes in the blood volume with each heart beat and various lower frequency components attributed to respiration, sympathetic nervous system activity, and thermoregulation.^[28]^

PPG was previously evaluated in labs to compare with PSG. PPG signals were used for detecting the changes in the volume of blood in the tissue microvascular bed by measuring the absorption of light from skin. Apnea and hypopnea were calculated based on ODI combined with a significant reduction of the PDR waveform. Barak-Shinar et al reported a similar sensitivity (94.4%) for AHI ≥5, but a much higher specificity (96.5%) than our results.^[31]^ Amir et al^[29]^ targeted on SDB patients with severe cardiovascular disease, and reported higher sensitivity (98%) and specificity (96%) of PPG measures compared to our results. Romem et al^[30]^ also found PPG-derived measure compared well with PSG in the diagnosis of suspected OSA among patients with and without cardiopulmonary comorbidities (for AHI < 5, PPG presented a sensitivity of 80%, a specificity of 86%, and for 15 ≤ AHI < 30, PPG presented a sensitivity of 70%, a specificity of 91%). Increased time of wakefulness or high ODI during sleep may cause the disagreement between PPG and PSG estimates, as the outliers that can be seen in Fig. 3.

In full PSG studies, TST was defined as the amount of actual sleep time from “light out” to “light on” and is calculated mainly based on manual electroencephalography scoring. In PPG monitoring, wake and sleep time were estimated based on the stability of the heart rate and breathing, and calculated according to epoch-by-epoch classification and total sleep duration for each study. Without position or actigraphy measurements, the accuracy of TST estimation may be compromised, which is a common problem for out of center sleep testing. However, estimation of total sleep time (TST) offers an advantage over some portable systems that use time in bed as the denominator for AHI. When sleep time calculation is compared, Romem et al^[30]^ reported that PPG failed to show any significant correlation between PSG derived total sleep time and PPG calculated total sleep time,^[30]^ while we found a moderate correlation between the 2 measures. Another study reported a regression coefficient of the PPG-TST yielded a result of 0.74, which is good considering the interscorer variability in the manual scoring of sleep time.^[31]^ The discrepancy could be due to the study sample size or heterogeneity of various demographic factors including OSA severity. Reliable estimation of total sleep time offers an advantage over some portable systems that use time in bed as the denominator for AHI. In this study, PPG-TST (324.0 ± 98.4 minutes) is shorter compared with PSG-TST (380.6 ± 74.0 minutes), suggesting that an improvement of PPG-derived TST estimation is needed. Meanwhile position or actigraphy measurements may provide additional values to detect the TST more accurately.

In addition to the number of induced electrodes attached to patients, the main difference between the PSG and PPG monitoring is that PPG does not require attended technician during the night or manual scoring after the night. Although the current study is conducted in a sleep laboratory environment, the system is expected to be used in home settings, and to allow the possibilities for simple operation, less resource-intensive, and cost-effective methods for OSA diagnosis or treatment follow-ups.^[36–39]^ To meet the demand of portable or home sleep studies, other alternative approaches have been proposed using different physiological signals.^[40–45]^ Examples include the devices using respiratory flow and/or respiratory effort, peripheral arterial tonometry,^[17,46]^ and actigraphy.^[47]^ The first 2 methods have been approved by the AASM for the OSA screening. Actigraphy is indicated to estimate TST in patients with OSA when PSG is not available.^[47]^ A combined use of actigraphy and respiratory monitoring allows the detection and elimination of wake time during the night, thus a more accurate AHI can be estimated.^[48]^ Another available Food and Drug Administration approved approach is based on ECG recordings during the night, called cardiopulmonary coupling analysis.^[49]^ Previous studies have proven such an ECG-based approach to be cost-effective and provide clinically useful insight into abnormal sleep in various patient populations^[50–53]^ by illustrating sleep states (stable or unstable sleep) in addition to the estimation of SDB. Each method or algorithm has their advantages and disadvantages. An appropriate combination of the methods for the targeted population can improve the diagnostic accuracy and provide complementary values in clinical interventions.

### 4.1 Limitations

We would like to acknowledge the following limitations of this study. First, this is a pilot study with a small sample size and no home monitoring is involved. Although we found significant association of PPG- and PSG-based measures, the absence of an evaluation at home settings obviates the possibility of stating definite conclusions regarding the effectiveness of PPG use at home. However, the good compliance of PPG monitoring at sleep lab and previous home-based studies using oximeters may support that PPG techniques can be used for home monitoring. Second, we only compared PPG measures with PSG outcomes. Further studies are encouraged to compare PPG with different portable monitors or alternative approaches for the use of home testing.

## 5 Conclusions

PPG derived measures have significant correlations with PSG measures. The overall sensitivity, specificity, PPV, and NPV indicated a good accuracy on detecting respiratory events from PPG recordings. The performance of PPG algorithm regarding automated calculation of REI, ODI, and TST illustrated reliable diagnostic values that are consistent with standard PSG measures. The utility of pulse oximeter system with PPG recordings allows the possibilities for simple operation, less resource-intensive, and cost-effective methods for OSA diagnosis or treatment follow-ups.
